# Importance of the putative furin recognition site _742_RNRR_745_ for antiangiogenic Sema3C activity *in vitro*

**DOI:** 10.1590/1414-431X20187786

**Published:** 2018-10-08

**Authors:** I. Valiulyte, V. Preitakaite, A. Tamasauskas, A. Kazlauskas

**Affiliations:** 1 Neuroscience Institute, Lithuanian University of Health Sciences, Kaunas, Lithuania; 2 Faculty of Medicine, Lithuanian University of Health Sciences, Kaunas, Lithuania

**Keywords:** Semaphorin, Sema3C, HUVEC, Angiogenesis, Furin-like pro-protein convertases

## Abstract

Angiogenesis is one of the key processes in the growth and development of tumors. Class-3 semaphorins (Sema3) are characterized as axon guidance factors involved in tumor angiogenesis by interacting with the vascular endothelial growth factor signaling pathway. Sema3 proteins convey their regulatory signals by binding to neuropilins and plexins receptors, which are located on the effector cell. These processes are regulated by furin endoproteinases that cleave RXRR motifs within the Sema, plexin-semaphorins-integrin, and C-terminal basic domains of Sema3 protein. Several studies have shown that the furin-mediated processing of the basic domain of Sema3F and Sema3A is critical for association with receptors. It is unclear, however, if this mechanism can also be applied to other Sema3 proteins, including the main subject of this study, Sema3C. To address this question, we generated a variant of the full-length human Sema3C carrying point mutation R745A at the basic domain at the hypothetical furin recognition site _742_RNRR_745_, which would disable the processing of Sema3C at this specific location. The effects produced by this mutation were tested in an *in vitro* angiogenesis assay together with the wild-type Sema3C, Sema3A, and Sema3F proteins. Our results showed that the inhibitory effect of Sema3C on microcapillary formation by human umbilical vein endothelial cells could be abrogated upon mutation at the Sema3C basic domain within putative furin cleavage site _742_RNRR_745_, indicating that this site was essential for the Sema3 biological activity.

## Introduction

Development of new blood vessels (angiogenesis) is one of the essential processes in the rapid growth, development, and metastasis of tumors. However, therapies used for treatment of tumor-associated angiogenesis are still limited and do not protect against recurrence of the tumor. Also, patients do not respond well because of the acquired drug resistance and toxicity (1). Therefore, a search for novel anti-cancer molecules with anti-angiogenic activity is needed. A recent review by Neufeld and coauthors (2) showed that class 3 semaphorins (Sema3) of the semaphorin protein family appear to be promising targets for strategies to prevent cancer progression.

Sema3 group, which consists of seven members (A–G), are secreted proteins responsible for axon guidance in the central nervous system. In addition, Sema3 regulate processes such as tumor growth, metastatic spread (2,3), and tumor-associated angiogenesis inhibition by interacting with vascular endothelial growth factor (VEGF) signaling pathway components (4,5). According to a review by Nasarre and coauthors (6), three of the seven Sema3 family proteins – Sema3D, Sema3F, and Sema3G – have tumor-suppressive and anti-angiogenic properties, and three others – Sema3A, Sema3B, and Sema3E – show promoting and inhibiting effects on the development of different types of cancer and angiogenesis processes. However, the role of Sema3C in tumor angiogenesis is controversial. Recent studies have shown that Sema3C is critical for gastric cancer angiogenesis (7); on the other hand, Sema3 acts as an inhibitor of pathological retinal angiogenesis (8). These different effects of Sema3C may depend on the stage of development of the tumor, the specificity of the tissue, and the Sema3C proteolytic processing.

Furin-like proteinases were shown to cleave Sema3 at different sites and thereby regulate their function (9). This statement was proven by several scientific studies, in one of which, for example, it was demonstrated that furin-like proteinases proteolytically activate the predominant form (p61) of Sema3E and promote tumor cell motility (10), whereas in other studies, the uncleavable form of Sema3E acts as anti-angiogenic and anti-metastatic factor (11,12).

Sema3B acts as a tumor suppressor in lung cancer and inhibits the formation of endothelial cells tubes in an *in vitro* angiogenesis; however, this function was abrogated upon mutation at the furin cleavage site (13).

Sema3 proteins possess several furin-recognition motifs RXRR that are found at the Sema domain, plexin-semaphorins-integrin (PSI) domain, and C-terminal basic domain (9). Several studies have revealed that proteolytic processing of Sema3C at the Sema domain (14) and PSI domain (8) had no significant effect in angiogenesis, indicating that other domains of Sema3C may be relevant for their function. Thus, our study was mainly focused on the putative furin recognition motif _742_RXRR_745_, which is located at the basic domain of Sema3C, since furin-cleavage motif(s) present in basic domains of Sema3A and Sema3F proteins were demonstrated to play an important role in mediating their association with neuropilin receptors (NRP1 and NRP2) and to have a competitive interaction with VEGF (15,16). To our knowledge, the importance of _742_RXRR_745_ motif for Sema3C function has not yet been analyzed; therefore, we generated a full-length variant of Sema3C carrying point mutation at the basic domain at the putative furin recognition site (_742_R745A_745_) and examined the effects of this mutation on Sema3C function in the angiogenesis process.

## Material and Methods

### Cell lines and chemicals

Human umbilical vein endothelial cells (HUVEC) (Cat. No. C-003-5C, Gibco, USA) were grown in endothelial growth cell media (M200, Gibco) with Low Serum Growth Supplement (LSGS, Cat. No. S00310, Gibco). Human embryonic kidney cells 293FT (Cat. No. R70007, Invitrogen, USA) were cultured in Dulbecco's modified eagle medium: nutrient mixture F-12 (DMEM/F-12) with 10 % fetal bovine serum (Gibco). All cell lines were incubated in a humidified atmosphere with 5% CO_2_ at 37°C.

### Plasmid construction and transfection

Bicistronic expression vectors encoding different semaphorins and the yellow fluorescing protein Venus, separated by IRES2 element, were constructed in two steps. First, we generated the intermediate construct pTO/IRES2-Venus by transferring the IRES2-Venus-coding BamHI-XbaI fragment from CSII-CMV-MCS-IRES2-Venus (kind gift of late Professor Lorenz Poellinger, Karolinska Institute, Sweden) to pcDNA4/TO plasmid (Invitrogen) at BamHI and XbaI restriction sites. In the second step, human Sema3A, Sema3F, and Sema3C-coding DNA fragments were generated by PCR using the following pairs of primers and respective templates: primers 5′-TCGGATCCATGGGCTGGTTAACTAGGATTG-3′ (forward), 5′-AGGGCACCCAGGAGTGTCTGAAGATCTTT-3′ (reverse) for Sema3A (template purchased from Dharmacon Inc., USA, accession: BC111416); 5′-CAGGATCCATGGCATTCCGGACAATTTGC-3′ (forward), 5′-TTGCGGCCGCCTATGACTCTGGCAACTGATTC-3′ (reverse) for Sema3C (template purchased from Ultimate ORF, ThermoFisher Scientific, USA, accession: NM_006379); and 5′-TCAGATCTATGCTTGTCGCCGGTCTTCTTCTC-3′ (forward), 5′-AAAGATCTTCATGTGTCCGGAGGGTGGTG-3′ (reverse) for Sema3F (template purchased from Dharmacon, accession: BC042914). The PCR products were digested with BamHI and BglII and ligated to pTO/IRES2-Venus opened with BamHI.

**Figure 1:**
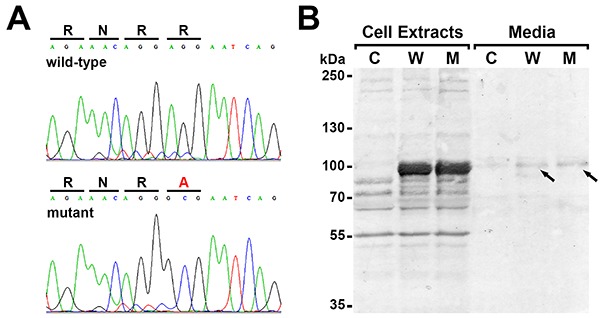
Verification of Sema3C R745A mutant expression and secretion. *A*, Sequencing results of the putative furin cleavage site _742_RNRR_745_ of the wild-type Sema3C and R745A mutant. *B*, Sema3C and R745A mutant protein expression in transfected 293FT cell extracts and cell medium. C: control (transfected with empty vector cells); W: wild-type Sema3C; M: mutant Sema3C (R745A). Black arrows indicate Sema3C and R745A mutant proteins of 85.2 kDa in size.

To generate the mutant Sema3C (R745A), the plasmid pTO/Sema3C-IRES2-Venus was modified using Phusion site-directed mutagenesis kit (ThermoFisher Scientific) and primers: 5′-GTAGAAACAGGGCGAATCAGTTGC-3′ (forward), 5′-TTTTCCGACTATTGATGAGGGCC-3′ (reverse). Modification of Sema3C at the furin cleavage site _742_RNRR_745_ was confirmed by sequencing (Figure 1A). 293FT cell transfection with different expression vectors was carried out using Lipofectamine 2000 reagent (Invitrogen), according to instructions of the manufacturer.

### Expression analysis of mRNA

Total RNA was extracted from transfected 293FT cells using PureLink RNA mini kit (Invitrogen) and the cDNA synthesis was performed with high capacity cDNA reverse transcription kit (Applied Biosystems, USA), according to the manufacturer's instructions.

The mRNA expressions of Sema3A, Sema3F, Sema3C, and Sema3C (R745A) were analyzed by reverse transcription PCR (RT-PCR) in a 50 µL reaction volume that consisted of 25 µL Maxima Hot Start Green PCR Master Mix (ThermoFisher Scientific), 10 ng cDNA of forward and reverse primers to a final concentration of 1 µM, and nuclease-free water. Primers for RT-PCR analysis of Sema3A, Sema3C, and Sema3C (R745A) expressions were the same as those used for cloning, except for Sema3F that were: 5′-CGATGACGGTGGTCACTGTTG-3′ (forward), 5′-CAGCGTAAATGACAGGGTTCCT-3′ (reverse, 169 bp amplicon size). In each set of RT-PCR analyses, two negative controls were used: nuclease-free water and cells expressing “empty” control vector pTO/IRES2-Venus. Amplification parameters for Sema3A, Sema3C, and Sema3C (R745A) were as follows: 95°C for 5 min, 25 cycles at 95°C for 20 s, 55°C for 30 s, 72°C for 2 min, and final 72°C for 7 min. Amplification parameters for Sema3F were 95°C for 5 min, 25 cycles at 95°C for 15 s, 58°C for 30 s, 72°C for 30 s, and final 72°C for 5 min.

### Protein analysis

The expression of Sema3A, Sema3F, Sema3C, and Sema3C (R745A) proteins in transfected 293FT cells and cell medium was analyzed with western blot. Cells were scratched off the plate in ice-cold phosphate buffered saline (PBS), suspended in RIPA lysis buffer (50 mM TrisHCl (pH 7.5), 150 mM NaCl, 1% Igepal CA-630, 0.5% sodium deoxycolate, 0.1% SDS) supplemented with protease inhibitor cocktail (ThermoFisher Scientific, Cat. No. 87786) and centrifuged for 40 min at 12.000 *g* at 4°C. Supernatant was collected and stored at –80°C. Simultaneously, semaphorin-containing and control cell medium from transfected 293FT cells was collected and concentrated to 20-fold with Pierce protein concentrators PES (ThermoFisher Scientific), according to the manufacturer's protocol. Eighty micrograms of each semaphorin protein and 20 µL of concentrated cell medium were loaded onto 7.5% SDS-PAGE and transferred to a nitrocellulose membrane. The membrane was blocked with 10% non-fat milk in PBS overnight followed by incubation with Sema3C rabbit polyclonal antibody (ThermoFisher Scientific, Cat. No. PA5-24997, dilution 1:500) for 2 h at room temperature. After washing with PBS-T buffer (PBS supplemented with 0.5% Tween-20), the membrane was incubated with secondary HRP-conjugated goat anti-rabbit antibody (Invitrogen, Cat. No. 65-6120, dilution 1:2000) for 40 min at room temperature. Protein signals were visualized using TMB substrate (Sigma-Aldrich, USA) and captured with the digital scanner.

### Endothelial tube formation assay

The 293FT cells were transfected with different semaphorin-encoding expression vectors and grown in M200 medium supplemented with low serum growth supplement (LSGS) for 48 h. Ice-cold Geltrex (40 µL) reduced growth factor basement membrane matrix (Cat. No. A1413201, Gibco) was added to a 96-well plate and incubated at 37°C for 30 min to polymerize. HUVEC cells (1.2 × 10^4^ per well) were suspended in 70 µL medium from transfected 293FT and seeded on top of Geltrex. Medium from transfected 293FT cells with pTO/IRES2-Venus was used as control. Three replicates of each protein were performed. After 2 and 16 h, the microcapillary structures were captured with a microscope (Lumascope LS620, Etaluma, USA) at 4 and 10× magnifications and analyzed with ImageJ angiogenesis analyzer program (U.S. National Institutes of Health, USA).

### Statistical analysis

The differences between the effects of Sema3A, Sema3F, Sema3C, and Sema3C (R745A) protein on endothelial tube formation were compared with two-tailed Student's *t*-test using GraphPad Prism software (version 5.0; GraphPad Software, Inc., USA). P<0.05 was considered to be significant. Data are reported as means ± standard deviation.

## Results

We constructed bicistronic expression vectors encoding Sema3A, Sema3F, or Sema3C proteins and fluorescent protein Venus separated by the IRES2 element. The R745A mutation in the basic domain of Sema3C within the putative furin cleavage site _742_RNRR_745_ was introduced by site-directed mutagenesis and verified by sequencing (Figure 1A). These plasmid constructions were used for transfection of the 293FT cells, which after 24 h exhibited fluorescence, indicating that the Sema3 expression vectors were successfully delivered into cells (Supplementary Figure S1A). To verify if the transfected cells synthesized Sema3 proteins (in addition to Venus), the mRNA expressions of Sema3A, Sema3F, Sema3C, and Sema3C (R745A) were analyzed using reverse transcription PCR (Supplementary Figure S1B). In order to examine whether the mutation R745A within the putative furin cleavage site has any effect on the integrity of Sema3C protein and its secretion to the extracellular milieu, we performed western blot analysis on extracts prepared from Sema3C and Sema3C R745A mutant-expressing cells and on growth media collected from these cells. Sema3C specific antibodies detected both the wild-type Sema3C and the mutant Sema3C (R745A) proteins in transfected 293FT cell extracts and culture medium as a protein of 85.2 kDa in size (Figure 1B). This result indicated that the R745A mutation did not alter Sema3C integrity (solubility and intactness) and its secretion to the growth medium.

The medium collected from transfected cells was used as a source of Sema3 proteins for examining effects of mutant Sema3C (R745A), with the comparison to the wild-type Sema3C, on microcapillary formation by HUVEC cells in *in vitro* angiogenesis assays. For this purpose, HUVEC cells were suspended in media containing secreted Sema3A, Sema3F, Sema3C, and Sema3C (R745A) and seeded on a 96-well plate coated with Geltrex. Because Sema3A and Sema3F are well known as angiogenesis inhibitors, these two proteins were used for comparative monitoring of Sema3C effects on the microcapillary tube formation. For control, HUVEC cells were seeded in medium, which was collected from cells expressing Venus protein alone.

**Figure 2:**
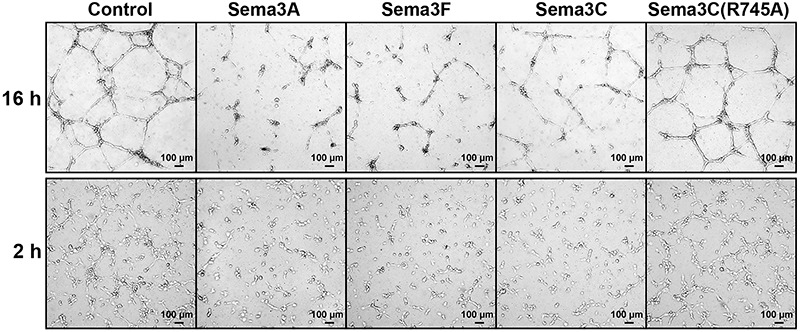
Effects of Sema3A, Sema3F, Sema3C, and Sema3C (R745A) proteins on microcapillary network formation by human umbilical vein endothelial cells after 16 and 2 h. Scale bar, 100 µm.

**Figure 3:**
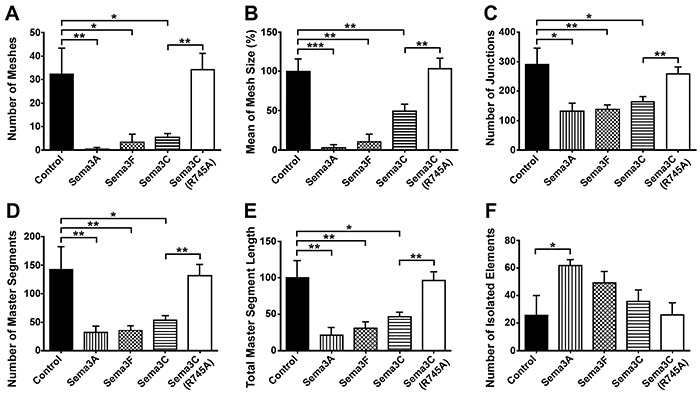
Analysis of microcapillary tube formation. Number of meshes (*A*), mean mesh area (*B*), number of junctions (*C*), number of master segments (*D*), total master segment length (*E*), and number of isolated elements (*F*) in Sema3A, Sema3F, Sema3C, and Sema3C (R745A) treatment and control groups. Data are reported as means±SD. *P<0.05; **P<0.01; ***P<0.001, two-tailed Student's *t*-test.

After 16 h, we observed completely formed microcapillary networks in control plates that were disintegrated in the presence of Sema3A and Sema3F proteins (Figure 2, upper panels). Noteworthy, Sema3C also evoked inhibition of the tube-like network formation, which was noticed in a recent study by Yang and colleagues (8). Importantly, the inhibitory effect of Sema3C was abrogated upon R745A mutation (Figure 2, upper panels). The corresponding effects of semaphorins, including the wild-type Sema3C and its mutant, were observed in much earlier stages of microcapillary network formation, e.g., after 2 h, as shown in the lower panels of Figure 2. The analysis of digital images of microcapillary networks was carried out using ImageJ angiogenesis analyzer software, with the help of which we estimated the number of meshes, mean mesh area, number of junctions, master segments, total master segment length, and number of isolated segments in all four Sema3 protein groups. The obtained data confirmed our visual observation that in the presence of Sema3A, Sema3F, and Sema3C, the parameters, which are associated with the HUVEC network integrity, were markedly reduced compared to control samples (Figure 3, panels A-E, P<0.05). Interestingly, the anti-angiogenic effects of Sema3C on some parameters such as mean mesh size and total master segment length (Figure 3, panels B and E, respectively) were less severe compared to, for example, Sema3A, which displayed the strongest inhibitory effect. Importantly, the network integrity-associated values registered from Sema3C R745A mutant samples resembled those of controls (Figure 3, panels A-E). The number of isolated segments was increased in various degrees upon treatment with Sema3A, Sema3F, and Sema3C proteins, whereas Sema3C R745A mutant, again, showed virtually no effect (Figure 3F). Thus, the results showed that Sema3C significantly inhibited the formation of a microcapillary network of HUVEC; however, this activity was lost upon R745A mutation, indicating that the C-terminal arginine of the putative furin cleavage site at the basic domain of Sema3C protein was critical for its functions in angiogenesis process.

## Discussion

Angiogenesis is one of the pivotal factors contributing to tumor development and growth, where VEGF is an essential regulator of tumor vessel formation. Thus, attention is being paid to finding novel molecules for targeting the VEGF signal pathway and control the tumor angiogenesis process (1). Recent scientific studies revealed that secreted class 3 semaphorins interact with the VEGF signaling pathway components and regulate angiogenesis processes (17). As shown for some of the semaphorins, these proteins can compete with VEGF due to its interaction with neuropilin receptors (NRP1 and NRP2), which leads to inhibition of endothelial cell migration, proliferation, and capillary structures formation of the tumor (17). With regard to the main subject of our study, Sema3C, the effects of this protein on angiogenesis processes remains controversial. Several studies showed that increased expression of Sema3C is a poor prognostic factor for patients with breast cancer (18), glioblastoma (19), or gastric cancer (7), implying that Sema3C may be involved in cancer progression possibly through the stimulation of angiogenesis (7,18). On the other hand, Sema3C was reported to inhibit pathological retinal angiogenesis by signaling via receptors NRP1 and Plexin D1 and by inducing endothelial cell apoptosis (8). In agreement with this, in the present study, we demonstrated that Sema3C significantly inhibited the microcapillary tube formation in an *in vitro* system. Therefore, depending on the specificity of the tissue, Sema3C could have pro-tumorigenic effect and induce tumor angiogenesis or, contrarily, act as an anti-angiogenic factor.

Malignant cells produce high levels of proteinases such as furin-like pro-protein convertases that could affect the Sema3C function by proteolytic processing. Sema3C could be cleaved by furin endoproteinases at consensus RXRR motifs, which are located in Sema, PSI, and basic domains. In the study of Mumblat and co-authors, a Sema3C variant resistant to furin cleavage at Sema domain RSRR site showed anti-tumorigenic effect by inducing contraction of lymphatic endothelial cells (LEC) and inhibiting the proliferation of LEC and HUVEC (14). This Sema3C mutant also inhibited tumor lymphangiogenesis and the metastatic spread of tumor cells to lymph nodes. Meanwhile, the cleaved form of Sema3C (p65-Sema3C) failed to induce the collapse of the cytoskeleton of LEC. However, the mechanism of p65-Sema3C is yet to be elucidated (14). Another study showed that wild-type Sema3C as well as 13 C-terminal amino acids lacking Sema3C isoform Sema3CΔ13 (imitating the processing by metalloproteinase at ALINS site) inhibited endothelial tube formation, elongation, and sprouting *in vitro*. However, short Sema3Cp60 isoform that resembles furin cleavage within the Sema3C PSI domain (RSRR site) was inactive (8). Remarkably, no detailed studies were done concerning the proteolytic processing of the basic domain of Sema3C. The importance of the furin-mediated processing of the basic domain of Sema3F and Sema3A, which results in exposure of C-terminal arginine of the RXRR motif at the end of the protein, was shown to be critical for association of these semaphorins with neuropilins and competition with VEGF (15,16). In order to elucidate this matter, we constructed a variant of Sema3C carrying point mutation (R745A) at the basic domain at the hypothetical furin recognition site _742_RNRR_745_, which would render Sem3C uncleavable at this particular location. Sema3C R745A mutant was expressed in cells and secreted into extracellular milieu as efficiently as the wild-type Sema3C; however, the inhibitory effects of Sema3C on formation of a tube-like HUVEC network were lost upon R745A mutation.

In summary, this study confirmed the Sema3C inhibitory effect of the angiogenesis process in an *in vitro* system (8) and for the first time demonstrated that Sema3C function may be abrogated upon mutation at the putative furin cleavage site of Sema3C, _742_RNRR_745_, indicating its importance for the Sema3C biological activity. Importantly, in the study of Parker and colleagues, it was shown that the C-terminal portion of Sema3F, which encompasses the furin cleavage site at the basic domain, interacts with Nrp2 only when C-terminal arginine of the RXRR motif is exposed (15). However, it is unclear if the R745A mutant is able to interact with the receptor neuropilin and if it is still able to activate signaling pathways (e.g., VEGFR and Rac1 activation pathways) within the cells. Zhu and coauthors demonstrated that the knockdown of SEMA3C significantly inhibited breast cancer cell MCF-7 growth and migration (20); therefore, it would be interesting to examine how the Sema3C R745A mutant affects the endothelial cell migration process. These studies would help to disclose the significance of the Sema3C protein furin cleavage site _742_RNRR_745_ in molecular processes.

## Supplementary material

Click here to view [pdf].
